# Interaction of spindle assembly factor TPX2 with importins-α/β inhibits protein phase separation

**DOI:** 10.1016/j.jbc.2021.100998

**Published:** 2021-07-21

**Authors:** Mohammad S. Safari, Matthew R. King, Clifford P. Brangwynne, Sabine Petry

**Affiliations:** 1 Department of Molecular Biology, Princeton University, Princeton, New Jersey, USA; 2 Department of Chemical and Biological Engineering, Princeton University, Princeton, New Jersey, USA; 3 Howard Hughes Medical Institute, Princeton University, Princeton, New Jersey, USA

**Keywords:** microtubule nucleation, spindle assembly factor, condensates, phase separation, IBB, importin-β-binding domain, NLS, nuclear localization signal, SEC-MALS, size exclusion chromatography in line with multiangle light scattering, TPX2, targeting protein for XKlp2

## Abstract

The microtubule-based mitotic spindle is responsible for equally partitioning the genome during each cell division, and its assembly is executed *via* several microtubule nucleation pathways. Targeting Protein for XKlp2 (TPX2) stimulates the branching microtubule nucleation pathway, where new microtubules are nucleated from preexisting ones within mitotic or meiotic spindles. TPX2, like other spindle assembly factors, is sequestered by binding to nuclear importins-α/β until the onset of mitosis, yet the molecular nature of this regulation remains unclear. Here we demonstrate that TPX2 interacts with importins-α/β with nanomolar affinity in a 1:1:1 monodispersed trimer. We also identify a new nuclear localization sequence in TPX2 that contributes to its high-affinity interaction with importin-α. In addition, we establish that TPX2 interacts with importin-β *via* dispersed, weak interactions. We show that interactions of both importin-α and -β with TPX2 inhibit its ability to undergo phase separation, which was recently shown to enhance the kinetics of branching microtubule nucleation. In summary, our study informs how importins regulate TPX2 to facilitate spindle assembly, and provides novel insight into the functional regulation of protein phase separation.

The propagation of life requires the rapid and accurate assembly of the microtubule-based mitotic spindle (1, 2). During mitosis, Ran is repurposed from its role in regulating nuclear import to serve as a key regulator of both microtubule nucleation and spindle organization (3, 4). At prometaphase, Ran gets converted into its GTP-bound state near chromatin, wherein it releases spindle assembly factors from sequestration by karyopherins (3, 5). There is a growing repertoire of about two dozen spindle assembly factors, but the molecular mechanism of how karyopherins inhibit spindle assembly factors, and thereby spindle assembly, is poorly understood (5, 6).

The majority of spindle assembly factors are inhibited by the canonical and abundant karyopherin complex, the importin-α/β heterodimer (6, 7, 8, 9, 10). Importin-α contains an importin-β-binding (IBB) domain, which in the absence of importin-β masks its nuclear localization signal (NLS) binding pocket and prevents the association of importin-α with NLS-containing proteins. Within the importin-α/β heterodimer, importin-β is bound to the IBB of importin-α, thereby making the heterodimer competent to bind the NLS-containing protein (11, 12, 13). It has been proposed that importin-α/β binding to NLS sites on spindle assembly factors sterically blocks microtubule binding domains that lie adjacent to the spindle assembly factor’s NLS (14, 15). However, the possibility of other modes by which importins could inhibit spindle assembly factors has yet to be explored (16, 17).

The spindle assembly factor and microtubule-binding protein targeting protein for XKlp2 (TPX2) (3) promotes the formation of spindle microtubules *via* branching microtubule nucleation (18), which contributes the majority of spindle microtubules (19, 20, 21). In this process, microtubules are autocatalytically amplified from preexisting ones, while preserving their polarity (18, 20). TPX2 is a key factor for branching microtubule nucleation, as it initiates this reaction by binding to microtubules and marking the branch site to recruit the other essential key molecules of this reaction (22, 23). Therefore, TPX2’s inhibition by importin-α/β is critical for the cell cycle and the onset of spindle formation. It was recently demonstrated that a fragment of TPX2 that localizes to microtubules *in vitro* overlaps with a known importin-α/β-binding site (23), which led to the proposal that importin-α/β sterically inhibits microtubule binding and thereby spindle assembly (14). However, importin-α/β reduces, but does not inhibit TPX2 microtubule localization *in vitro* (24) and does not appear to affect microtubule localization in isolated *Xenopus* egg cytosol (12, 25). Furthermore, the minimal functional fragment of TPX2 for branching microtubule nucleation does not contain this microtubule-binding region yet can bind to microtubules (26). Finally, the molecular architecture of the TPX2-importin–α/β complex and their binding affinity remain unknown. Therefore, we investigated how importin-α/β inhibits TPX2 and thereby branching microtubule nucleation.

Proteins can condense, often *via* liquid–liquid phase separation, to achieve compartmentalization or reaction enhancement (27) for a range of cellular processes, including spindle assembly (27, 28, 29, 30). Regulation of condensates and their associated functions is therefore crucial for cellular health. A karyopherin related to importin-α/β (karyopherin 2-β) was recently shown to prevent aberrant cytoplasmic condensation of nuclear proteins by engaging in weak interactions distributed throughout the karyopherin and target protein (16, 17, 31). We recently uncovered that TPX2 undergoes condensation *via* liquid-liquid phase separation to enhance the reaction efficiency of branching microtubule nucleation (32). Most importantly, importin-α/β inhibits TPX2 phase separation *in vitro* and TPX2-mediated microtubule nucleation in isolated *Xenopus* cytosol (32). However, the molecular details of how importins inhibit TPX2 condensation and function remain unknown.

Here, we characterize how importin-α/β interacts with TPX2 and determine which interactions are relevant for inhibiting TPX2 condensation and function. We identified a new NLS within TPX2 that interacts with importin-α and demonstrate that importin-β inhibits TPX2 condensation by engaging in dispersed, weak interactions. Similarly, we demonstrate that these same weak interactions are sufficient to inhibit TPX2-mediated branching microtubule nucleation. These findings highlight a critical role for dispersed, weak interactions in the inhibition of an essential spindle assembly factor by karyopherins and may also inform how other phase separating proteins are regulated.

## Results

### Importin-α or importin-β alone inhibit TPX2-mediated branching microtubule nucleation

**Figure 1:**
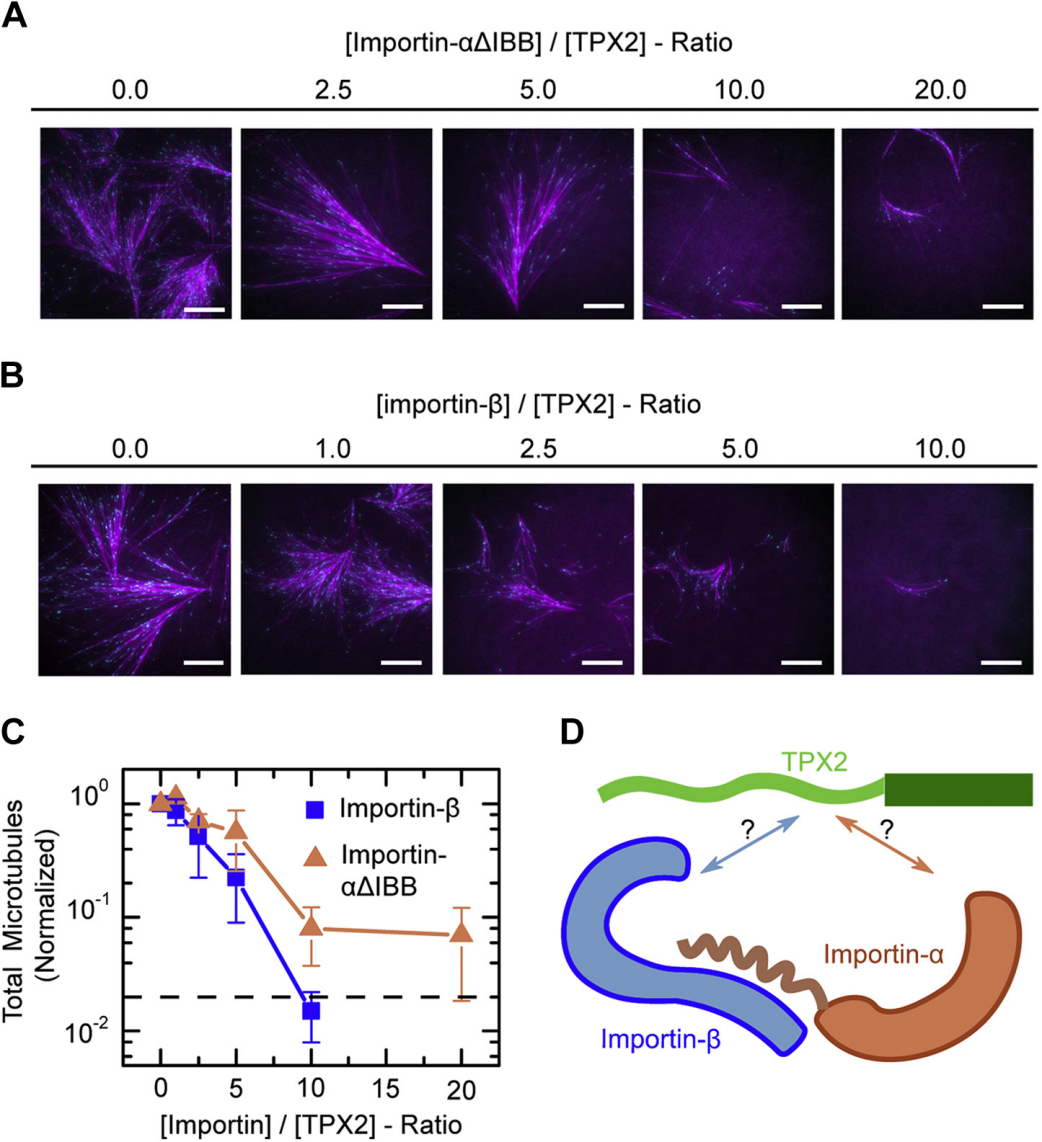
**Competency of importins at inhibiting TPX2-mediated branching microtubule nucleation in cytosol.***A* and *B*, representative total internal reflective fluorescence micrographs of TPX2-mediated branching microtubule nucleation in *Xenopus* meiotic cytosol in the presence of importin-αΔIBB (*A*) or importin-β (*B*) at indicated fold excess and concentrations. [TPX2] was kept constant at 150 nM and importins were added in excess 2.5× (375 nM), 5× (750 nM), 10× (1.5 μM), or 20× (3 μM). Images were taken 20 min into the reaction. Cy5-labeled tubulin (*magenta*) and mCherry-EB1 (*cyan*) highlight microtubules and growing microtubule plus ends, respectively. *C*, total number of microtubules generated by 20 min at indicated fold-excess concentration of importin-αΔIBB (*dark orange*) or importin-β (*blue*). Each data point represents the total number of microtubules in an analyzed field of view, which is normalized to the no importin condition from that experimental set. The *dashed line* represents total microtubules nucleated after 20 min with no proteins added (*i.e.*, background). *D*, schematic of TPX2–importin-α/β heterodimer. In the importin-α/β complex, the nuclear localization sequence–binding pockets of importin-α are exposed upon binding of importin-β to the IBB (importin-β-binding) domain of importin-α (a.a. 1–70).

In classic work using *Xenopus* egg cytosol, TPX2 was demonstrated to be the key factor downstream of RanGTP that initiates the formation of microtubule networks (3, 33). The nucleation capacity of both RanGTP and TPX2 can be suppressed by adding excess importins to *Xenopus* egg cytosol (3, 33), but the molecular nature of this effect remained unclear. Using total internal reflective fluorescence microscopy to resolve microtubule nucleation events, it was recently shown that TPX2, when added to *Xenopus* egg extract, induces the formation of branched microtubule networks (18) (Fig. 1*A*). Previously, we used this method to reveal that the importin-α/β heterodimer inhibits TPX2-mediated branching microtubule nucleation (32). To decipher the role of each importin subunit within this inhibition process, we tested whether importin-α or importin-β alone suppresses TPX2-mediated branching microtubule nucleation.

In the importin-α/β heterodimer, importin-β binds to importin-α’s autoinhibitory IBB domain to expose the NLS-binding pocket on importin-α. To mimic this state, we used a truncated form of importin-α, which does not contain the IBB domain (importin-αΔIBB) (11, 24) (Figs. 1*D*). We added TPX2 mixed with either importin-αΔIBB or importin-β to *Xenopus* egg cytosol, keeping TPX2 constant at 150 nM and including importins at a range of fold excesses—2.5× (375 nM), 5× (750 nM), 10× (1.5 μM), or 20× (3 μM). When only importin-α-ΔIBB was included at 20-fold molar excess of TPX2 the total number of microtubules nucleated relative to the no importin condition was drastically reduced (Fig. 1, *A* and *C*). Furthermore, TPX2 addition with only importin-β at 10-fold molar excess led to an even greater reduction in microtubule number than importin-αΔIBB at 20-fold molar excess, with branching microtubule nucleation being reduced to background levels (Fig. 1, *B* and *C*). These data show that, surprisingly, importin-α and importin-β can each independently inhibit TPX2-mediated branching microtubule nucleation, with importin-β being particularly effective. Next, we investigated the nature of the interactions between TPX2 and importin-α or importin-β to gain mechanistic insight into this inhibition.

**Figure 2:**
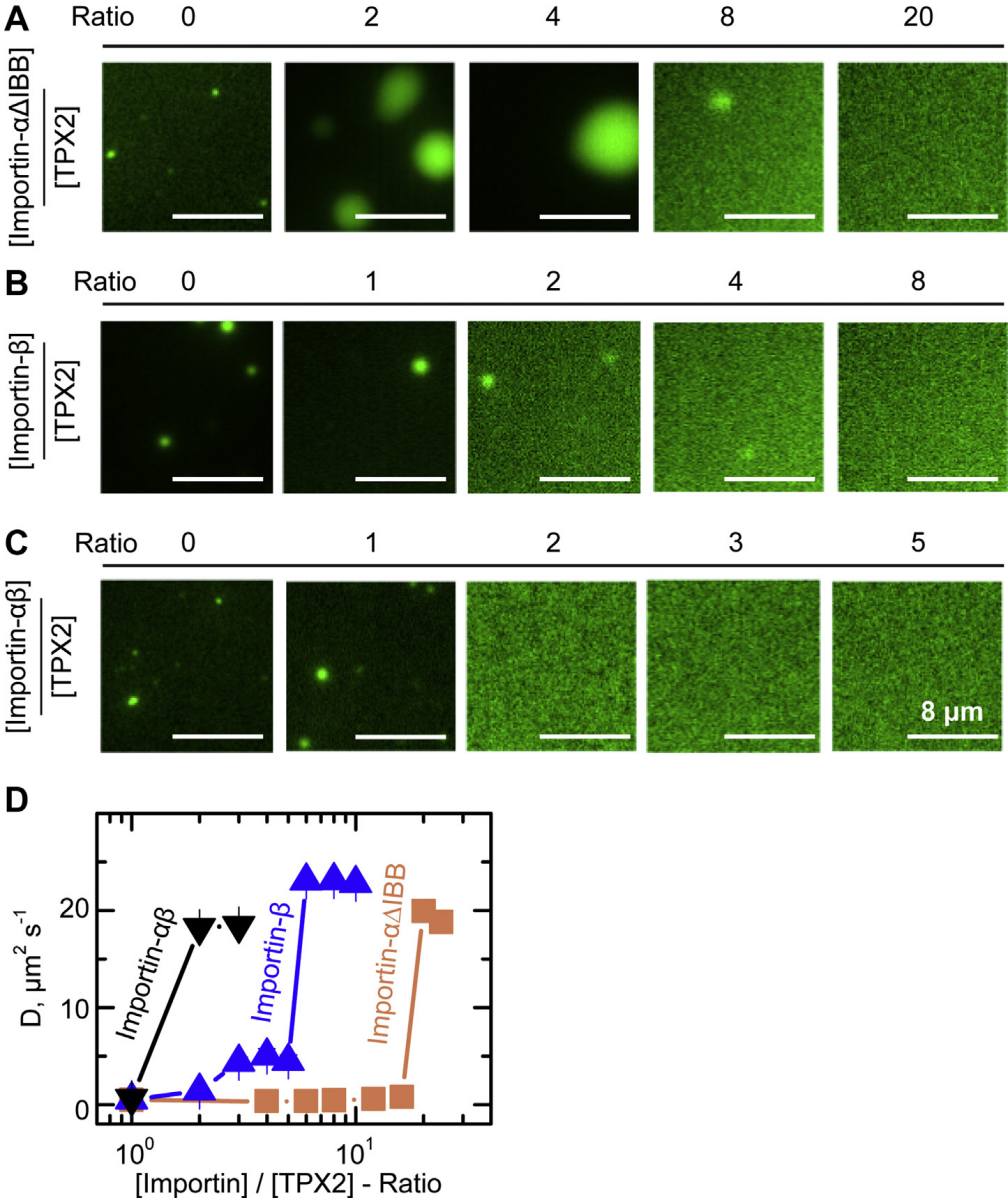
**Competency of importins at inhibiting TPX2 condensation.***A*–*C*, representative epifluorescence microscopy images of TPX2 in the presence of (*A*) importin-αΔIBB, (*B*) importin-β, and (*C*) importin-α/β at indicated concentrations and fold excess of importin to TPX2. [TPX2] was kept constant at 1 μM, and importins were added in excess 1× (1 μM), 2× (2 μM), 3× (3 μM), 4× (4 μM), 5× (5 μM), 8× (8 μM), or 20× (20 μM). *D*, diffusion coefficient of TPX2 solutions in CSF buffer (+0.1 M KCl) in the presence of indicated ratio of importin-αΔIBB, importin-β, or importin-α/β to TPX2 concentration as measured by dynamic light scattering. The error bars are calculated from ten distinct intensity–intensity correlation functions. TPX2 concentration is fixed at 1 μM.

**Figure 3:**
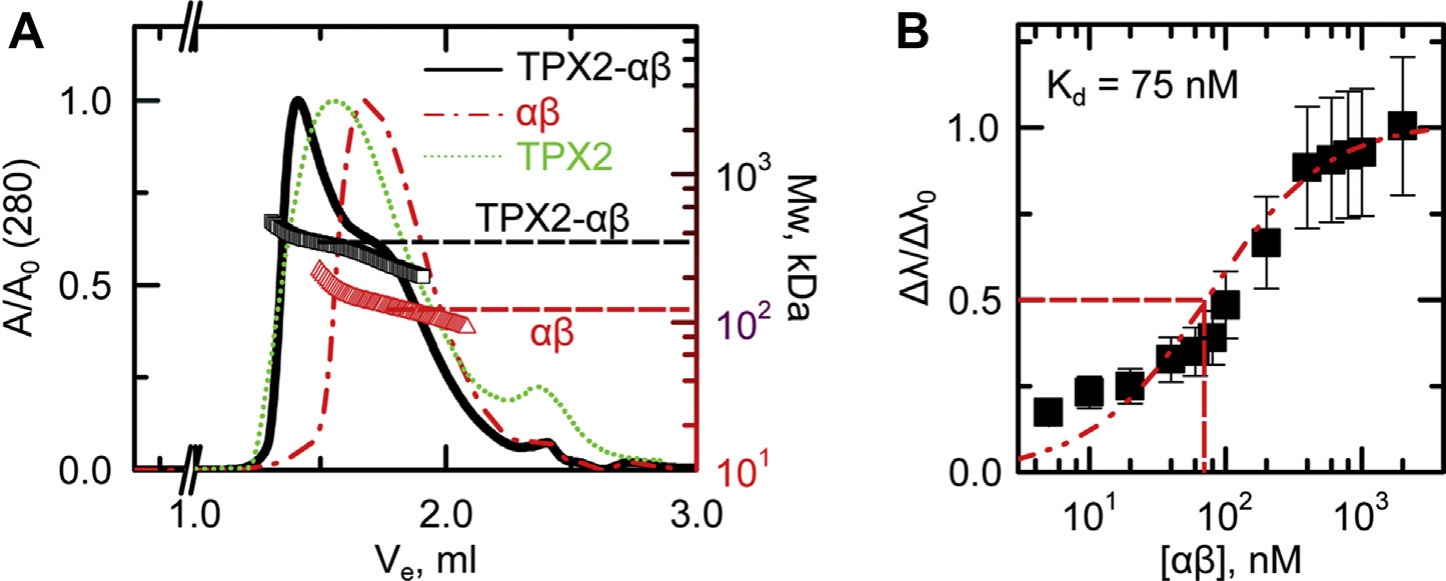
**TPX2 strongly associates with importin-α/β to form a trimer.***A*, size exclusion chromatography in line with light scattering reveals a stoichiometry of 1:1 for importin-α/β and 1:1:1 binding for TPX2–importin-α/β trimer. The *left axis* is normalized absorbance (λ = 280 nm), and the *right axis* shows the molecular weight of eluted complexes. The molecular weight of eluted complex for TPX2–importin-α/β and importin-α/β is shown with *black* (310 kDa) and *red* (130 kD) *arrows*, respectively. The molecular weight of TPX2 alone (180 kDa) is shown in Fig. S1*A*. TPX2 and importin-α/β concentrations are 5 M and 15 μM, respectively. *B*, biolayer interferometry (Octet) normalized amplitude as a function of importin-α/β concentration. The measured binding constant is Kd = 75 ± 15 nM. The error bars are calculated from two distinct measurements. At the low concentrations, the OCTET readout is approaching the limit of detection (<0.1).

**Figure 4:**
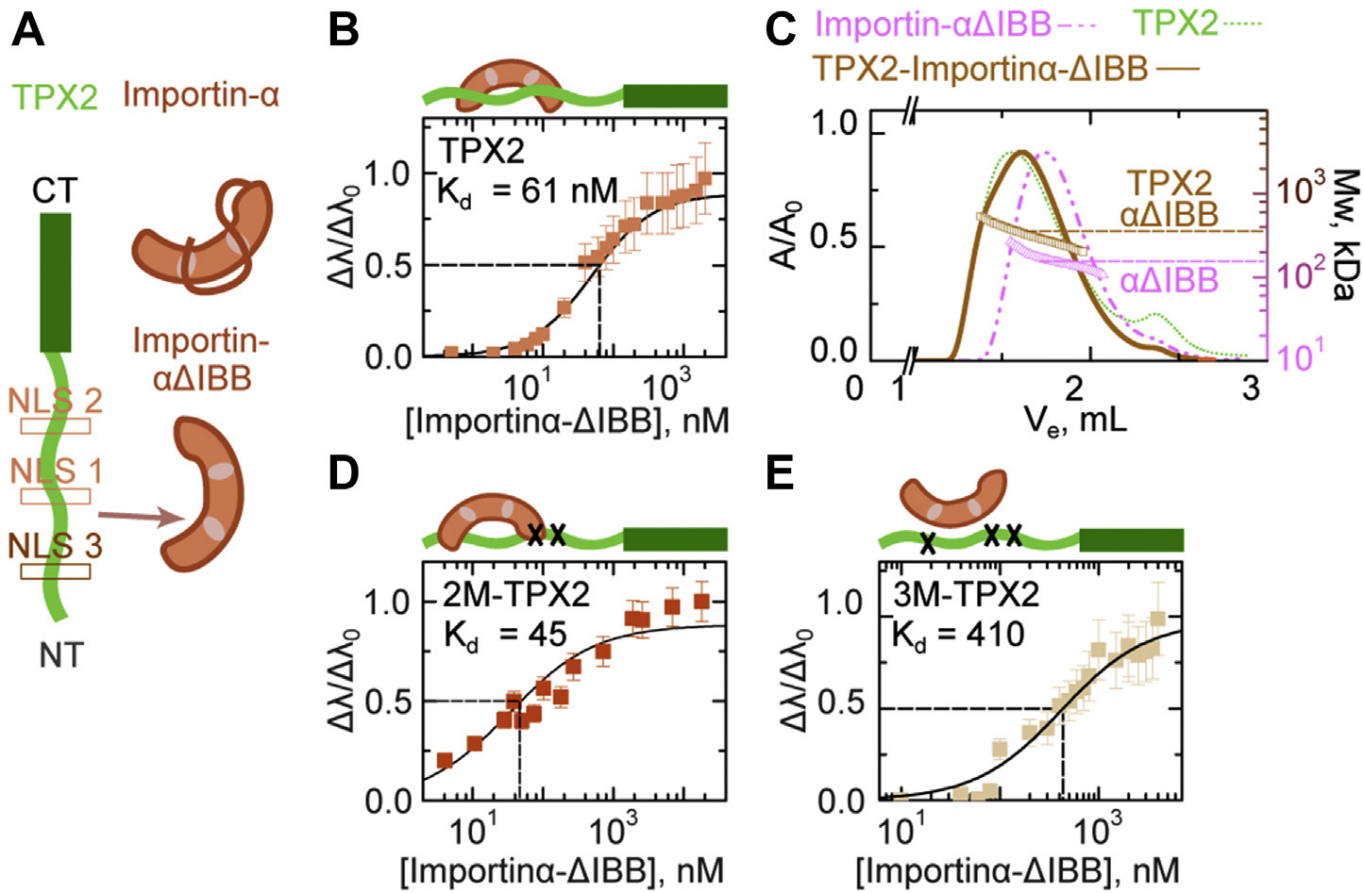
**TPX2 interacts with importin-αΔIBB *via* nuclear localization sequences at a.a. 123 and a.a. 284.***A*, the architecture of TPX2, importin-α, and importin-αΔIBB. TPX2 comprises a disordered N-terminal region (*light green*, a.a.1–480) and an ordered C-terminal region (*dark green*, a.a. 480–716). The two previously reported nuclear localization sequences (NLSs) are shown in *light brown*, centered at NL1 a.a. 284 and NL2 a.a. 327. The newly identified putative NLS3 is shown in *brown*, centered at a.a. 124. Importin-α, in the absence of importin-β, exists in an autoinhibited conformation wherein the NLS-binding pockets are occluded by the IBB domain. A truncated version of importin-α without the IBB domain (importin-αΔIBB) has exposed NLS-binding pockets. *B*, TPX2 binding to importin-αΔIBB is mediated by NLSs at a.a. 124 and a.a. 284. Biolayer interferometry (Octet) of wild-type TPX2 with importin-αΔIBB. Wild-type TPX2 and importin-αΔIBB associate strongly with K_d_ = 61 ± 10 nM. *C*, TPX2 interacts with importin-αΔIBB as a dimer/trimer complex. Size exclusion chromatography in line with light scattering reveals a stoichiometric 2:1/1:2 and 1:1 binding for importin-αΔIBB with TPX2. The *left axis* is normalized absorbance (λ = 280 nm), and the *right axis* shows the molecular weight of the eluted complex. The TPX2 and importin-αΔIBB concentrations are 2 and 20 μM, respectively. *D* and *E*, biolayer interferometry (Octet) of double mutant TPX2 (2M-TPX2) and triple-mutant TPX2 (3M-TPX2) with importin-αΔIBB, respectively. 2M-TPX2 for which the two NLSs (lysines at 284/5/7, and 325/8 are mutated to alanines) and importin-αΔIBB associate strongly with K_d_ = 45 ± 6 nM. Triple-mutant TPX2 (3M-TPX2), for which the three NLSs (lysines at 123/4/6, 284/5/7, and 325/8) are mutated to alanines, exhibits a 10-fold lower binding affinity to importin-αΔIBB, K_d_ = 410 ± 70 nM. Values are normalized amplitudes as a function of importin-αΔIBB concentration. Error bars are calculated from two distinct measurements.

### Importin-α or importin-β alone inhibit TPX2 condensation

TPX2 binding to microtubules serves as the first essential step to build a branch site, from which TPX2 recruits additional branching factors (22, 23). Moreover, we showed that TPX2 forms a liquid-like co-condensate with tubulin on the microtubule lattice, which enhances the reaction kinetics of branching microtubule nucleation (32, 34). Thus, the ability to inhibit TPX2 condensation could be key to the regulation of branching microtubule nucleation and the onset of spindle assembly. Therefore, we investigated the role of individual importins and the importin-α/β heterodimer in inhibiting TPX2 condensation.

We monitored TPX2 condensation *via* fluorescence microscopy with the inclusion of either importin-αΔIBB or importin-β alone or the importin-α/β heterodimer. TPX2 concentrations were held constant at 1 μM, and importins were included at a range of molar excess concentrations. Mixtures of importin-αΔIBB and TPX2 resulted in an enhancement of TPX2 condensation at lower concentrations followed by an inhibition of TPX2 condensation at approximately 20-fold excess (Fig. 2*A*). In contrast, importin-β inhibited TPX2 condensation between 4- and 8-fold excess and importin-α/β did so at 2-fold excess, making them both more effective than importin-α (Fig. 2, *B* and *C*).

To ensure that importin-inhibited TPX2 solutions are indeed monodisperse and do not contain any subresolution condensates, we tested each sample with dynamic light scattering, which measures an intensity–intensity autocorrelation function of scattered light from the solution. Because the scattered intensity is proportional to the sixth power of particle sizes, any residual amount of condensate can be detected. Our light scattering data further validated the results obtained by light microscopy: importin-α/β abrogates TPX2 condensation more efficiently than importin-β (Fig. 2*D*). Importin-β, in turn, suppressed TPX2 condensation more efficiently than importin-αΔIBB. The efficiency of inhibiting TPX2 condensation is correlated with importin-β’s ability to inhibit branching microtubule nucleation more strongly than importin-αΔIBB (Fig. 1, *A*–*C*). Yet, this was surprising because, although TPX2 has an NLS that is known to interact with importin-α, it does not harbor any known IBB sites. Therefore, we next interrogated how TPX2 interacts with the importin-α/β heterodimer, as well as importin-αΔIBB and importin-β alone (Fig. 1*D*).

### TPX2 strongly associates with importin-α/β to form a trimer

Although small fragments of TXP2 have been shown to bind to importin-αΔIBB (12, 24), it remained to be tested how full-length TPX2 associates with the importin-α/β heterodimer (Fig. 1*D*). We first addressed this question by determining the molecular weight and stoichiometry of the TPX2–importin-α/β complex *via* size exclusion chromatography in line with multiangle light scattering (SEC-MALS) (Fig. 3*A*). GFP-TPX2 eluted off the SEC column with an average molecular weight of 159 ± 52 kDa, comparable with its predicted molecular weight of 112.1 kDa, indicating that it exists mainly as a monomer with no detectable aggregate/oligomers (Fig. 3*A*, green dotted curve and Fig. S1*A*).

We then allowed GST-importin-α to bind to importin-β and assessed the formation of the importin-α/β heterodimer *via* SEC-MALS. The eluted complex exhibited a molecular weight of 130.0 ± 29 kDa determined *via* MALS, consistent with the predicted molecular weight of the importin-α/β heterodimer in a 1:1 stoichiometry (165.0 kDa) (Fig. 3*A*, red dashed curve and Fig. S1*B*).

When TPX2 and importins-α/β were allowed to bind, they eluted as a complex faster than TPX2 and importins-α/β alone. This rapid elution reflects a larger molecular weight of 310 ± 70 kDa *via* MALS (Fig. 3*A*, black solid curve) similar to the predicted molecular weight of 286.8 kDa. This suggests that TPX2–importin-α/β exists as a trimer with 1:1:1 stoichiometry.

In order to determine how strongly TPX2 interacts with importin-α/β, we determined their equilibrium dissociation constant *via* biolayer interferometry, which detects refractive index fluctuations upon binding and dissociation events of target proteins on the sensor surface (13, 34). We determined a K_d_ between TPX2 and importin-α/β of 75 ± 15 nM (Fig. 3*B*), indicating that TPX2 binds strongly to importin-α/β.

### TPX2 interacts strongly with importin-α *via* nuclear localization sequences

We next investigated how each individual importin binds to TPX2, both to address how importin-α and importin-β each contribute to forming the TPX2-importin-α/β trimer and to interrogate how their respective binding mode relates to their ability to inhibit TPX2.

By applying biolayer interferometry, we measured a dissociation constant of K_d_ = 61 ± 10 nM between importin-αΔIBB and TPX2 (Fig. 4*B*), indicating it alone has a strong affinity for TPX2, comparable with importin-α/β. We next asked whether this interaction is mediated by the two nuclear localization sequences (NLSs), NLS1 and NLS2, which had previously been shown to form a complex with importin-αΔIBB (Fig. 4*A*) in a crystal structure (23). This crystal structure was obtained with a co-complex of importin-αΔIBB and a small fragment of *Xenopus* TPX2 (a.a. 270–350). It was demonstrated that NLS1 solely mediates the interaction with importin-αΔIBB, whereas NLS2 is dispensable and not conserved to humans (23). We created a double-mutant TPX2 (2M-TPX2), in which NLS1 and NLS2 were mutated to alanines at the key residues that mediate this interaction (in NLS1 K284A and R285A, and in NLS2 K327A and K330A, were mutated). Surprisingly, the 2M-TPX2 exhibited a similar binding affinity to importin-αΔIBB as wt-TPX2 (wild-type TPX2), with a dissociation constant K_d_ = 45 ± 6 nM (Fig. 4*D*). This suggests that another strong binding site on TPX2 must exist to allow importin-αΔIBB to bind.

To investigate where this potential new binding site was located, we first assessed whether it lies within the C-terminal half of TPX2 (amino acids 319–716). However, this construct exhibited an extremely weak association with importin-αΔIBB (Fig. S2), suggesting that an additional binding site must exist within TPX2’s N-terminal half (amino acids 1–318). Because NLS2 lies within TPX2’s C-terminal half, this finding corroborates that NLS2 does not contribute to the interaction of TPX2 with importin-αΔIBB (23).

Since NLS1 and NLS2 were previously identified by scanning TPX2 amino acids 270 to 350 (23), we focused the search for a new binding site to amino acids 1 to 260 of TPX2. We created three TPX2 constructs that cover amino acids 1 to 99, 1 to 178, and 1 to 260. The short construct 1 to 99 exhibited only weak binding to importin-αΔIBB, whereas the two longer TPX2 constructs 1 to 178 and 1 to 260 exhibited strong binding with dissociation constants of 15 ± 3 and 20 ± 4 nM, respectively (Fig. S2). Bioinformatic analysis revealed a putative NLS sequence within TPX2, in amino acids KKLK located at positions 123 to 126, which is conserved in mammals. To test whether this sequence indeed functions as an NLS, we mutated the lysines to alanines within this motif of TPX2 1 to 178 (K123A, K124A, K126A), which resulted in a 10-fold loss of binding to importin-αΔIBB compared with the wild-type-1-178 TPX2 construct (Fig. S2). Furthermore, it was shown that the comutation of NLS1 and NLS3 led to a more significant reduction in nuclear import in mammalian cells than mutation of either alone (35). These findings indicate that the KKLK motif (amino acids 123–126) of TPX2 constitutes another NLS sequence, which facilitates TPX2 binding to importin-αΔIBB and import into the nucleus, and which we, therefore, term NLS3 (Fig. S2).

To assess whether NLS1 and NLS3 are indeed the major and only bindings sites on TPX2 for importin-αΔIBB, we created a triple-mutant TPX2 (3M-TPX2), for which all the NLS sequences were modified (K123A, K124A, K126A, K284A, R285A, K327A, and K330A). Strikingly, 3M-TPX2 exhibited a 10-fold decrease (K_d_ = 410 ± 70 nM) in the strength of its interaction with importin-αΔIBB, compared with 2M-TPX2 or WT-TPX2 (Fig. 4, *B* and *D*). This suggests that, within the entire sequence of TPX2, NLS1 and NLS3 represent the only canonical NLS sites that promote high-affinity interactions with importin-αΔIBB.

Because TPX2 contains two NLS sequences to which importin-αΔIBB can bind, we investigated the stoichiometry of the TPX2-importin-αΔIBB complex *via* SEC-MALS. The eluted complexes exhibited a molecular weight of 326 kDa with a dispersity of ±88 kDa (Fig. 4*C*). Comparison of the TPX2–importin-αΔIBB MALS profile to importin-αΔIBB alone, which displays a molecular weight of 159 kDa (implying it dimerizes), suggests TPX2–importin-αΔIBB is relatively dispersed and exists in a complex with stoichiometries above 1:1 (Fig. 4*C*). Taken together, these data show that importin-αΔIBB strongly interacts with TPX2 *via* two NLS sequences.

### TPX2 weakly and reversibly associates with importin-β

**Figure 5:**
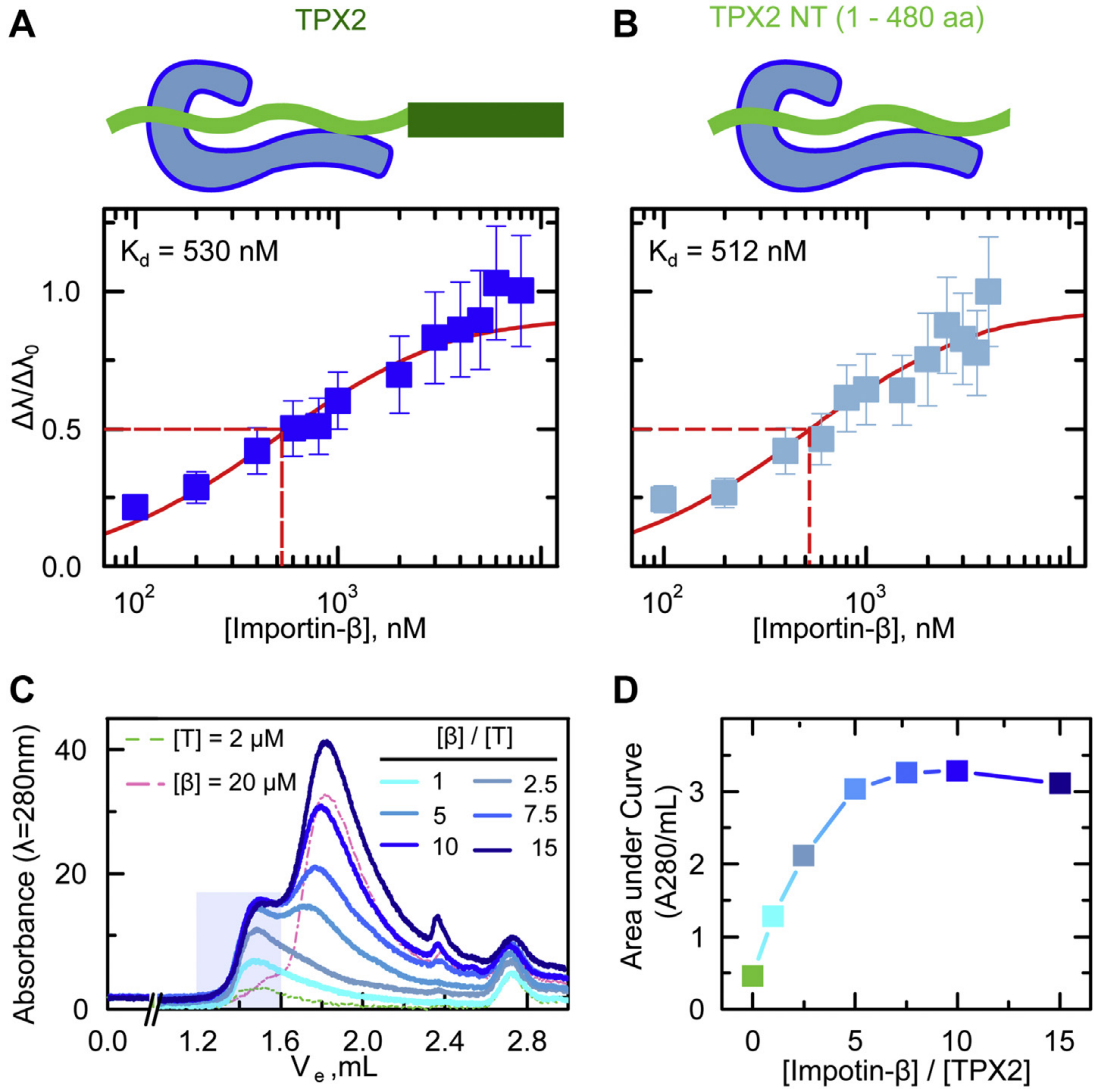
**TPX2 weakly and reversibly associates with importin-β.***A* and *B*, importin-β weakly associates with TPX2-N terminus. Biolayer interferometry (Octet) measuring the association of importin-β with (*A*) full length TPX2, K_d_ = 530 ± 75 nM, and (*B*) the N-terminal region (a.a. 1–480) of TPX2, K_d_ = 512 ± 70 nM. Values are normalized amplitude as a function of importin-β concentration. Error bars are calculated from two distinct measurements. *C* and *D*, the TPX2–importin-β complex is reversible. *C*, size exclusion chromatography with TPX2–importin-β complex exhibits dissolution of the complex upon varying the importin-β concentration under constant TPX2 concentration. The *left axis* is absorbance (λ = 280 nm). The *dashed green* and *pink* chromatograms are TPX2 and importin-β alone at concentrations 2 and 20 μM, respectively. *D*, area under the curve in the complex elution area (1.2–1.6 ml-A280) exhibits an equilibrium saturation for varying concentrations of importin-β.

Importin-β is known to associate with some nuclear proteins (8) and affect aspects of spindle assembly (36) independent of importin-α. Given that importin-β can strongly suppress both TPX2 phase separation and TPX2-mediated branching nucleation (Figs. 1 and 2), we reasoned that importin-β could associate with TPX2 in the absence of importin-α, rather than solely acting as an inert adaptor to occlude the IBB domain of importin-α, according to the existing model. To test this, we measured the binding affinity between TPX2 and importin-β by biolayer interferometry. Surprisingly, importin-β and TPX2 only weakly associate, with a dissociation constant of K_d_ = 530 ± 75 nM. Of interest, this association is driven by TPX2’s N terminus (a.a. 1–480), which exhibits a similar dissociation constant of 512 ± 70 nM (Fig. 5*B*) as full-length TPX2. Meanwhile, TPX2’s C terminus (a.a. 480–716) displays almost no binding with importin-β, exhibiting a K_d_ > 4 μM (Fig. S1*D*).

Given the surprisingly weak interactions between TPX2 and importin-β, we next examined the stoichiometry of the TPX2–importin-β complex. The SEC-MALS data displayed complexes eluting in the range of 200 to 700 kDa, indicative of a range of oligomeric species (Fig. S1*C*), which could be due to reversible assembly or irreversible aggregation. To delineate between these possibilities, we conducted a series of SEC experiments keeping TPX2 at 2 μM and varying importin-β concentration ranging from 2 to 30 μM, and integrated the area under the peak corresponding to the eluted complexes from SEC-MALS (blue shading, Fig. 5*C*). If these complexes were irreversible aggregates, a linear dependence on importin-β concentration would be expected. Instead, we observed a sigmoidal binding curve (Fig. 5*D*) suggesting that there exists a dynamic equilibrium that saturates upon the addition of importin-β at 3-fold excess. To confirm that the saturation is not due to consumption of TPX2, we calculated the unbound fraction of TPX2 using the dissociation constant of K_d_ = 0.5 μM measured by biolayer interferometry. At the maximum concentration of importin-β, c = 30 μM, more than 5% of TPX2 is still in the unbound state. Based on these measurements, the concentration of importin-β would need to exceed 100 μM for ≥99% of the 2 μM of TPX2 to be bound. Therefore, the saturation of the SEC curve was not likely due to the depletion of TPX2 and rather due to the equilibrium nature of the TPX2:importin-β complex. Thus, our data demonstrate that TPX2 weakly and reversibly associates with importin-β and this appears to be sufficient to prevent TPX2 phase separation (Fig. 2).

Last, we sought to explore explanations for why importin-β is effective at inhibiting TPX2 phase separation and its branching microtubule nucleation function, despite a relatively weak binding. The low pI of importins (pI < 5.5) and the high negative surface charge suggest that the proteins should be self-repulsive (Fig. S3*A*), which could be a mechanism for importins to prevent TPX2 phase separation when they bind. To address this possibility, we quantified the osmotic compressibility of importin solutions *via* the second virial coefficient (B_2_) obtained from static light scattering, wherein repulsion would result in a positive B_2_ (37). Strikingly, importins exhibited a negative B_2_ (slope in Fig. S3*C*), indicative of attractive intermolecular van der Waals forces originating from the exposed hydrophobic residues on the surface of importins (Fig. S3*B*) (37, 38). Despite these attractive forces, we do not observe macromolecular phase separation of importins in bulk solution, suggesting that we are below the critical saturation concentration.

Of most interest, the magnitude of B_2_, and therefore the strength of intermolecular van der Waals forces, follows the competency of importins to impede the condensates—$\left|B_{2}\;\right|$: importin-α/β > importin-β > importin-αΔIBB. This correlation suggests that short-range van der Waals forces play an important role in disrupting TPX2 condensation, which may work in conjunction with or supersede electrostatic repulsion of TPX2–importin complexes. Furthermore, these observations suggest that van der Waals forces probably do not contribute to a high-affinity interaction, which appears to be confined to TPX2’s NLSs. The weak nature of van der Waals interactions could explain why excess molar amounts of importins were required to inhibit TPX2 condensation, similar to the excess molar of Karyoperhin-2β needed to inhibit fused in sarcoma condensation (16, 31). Future experiments are needed to determine the role that van der Waals interactions play in preventing condensation of TPX2 or other karyopherin-regulated proteins.

## Discussion

Here we investigated the mechanism of TPX2 regulation by importins-α/β. Previous structural studies indicated that inhibition of TPX2 function may be achieved by importin-α directly blocking a microtubule-binding region of TPX2 (14, 24). Yet, this mechanism appears to be incomplete since importin-α/β only partially reduces TPX2 localization to microtubules *in vitro* and does not appear to reduce binding in cytosol (24, 25). Furthermore, the microtubule nucleation function of TPX2 resides in a C-terminal region of TPX2 that lacks the putative microtubule localization domain (18).

The stoichiometry of a full-length spindle assembly factor bound to importin-a/b had not been determined before but was only inferred from studies of non-spindle assembly factors, such as the classic nuclear protein nucleoplasmin (11), and a truncation of the spindle assembly factor Nuclear Mitotic Apparatus protein 1 (NuMA) (15). It was not evident *a priori* that TPX2 would engage with importin-α/β as a trimer as inferred, since TPX2 is ∼4× longer than nucleoplasmin, mostly disordered (≥70%) (26), and associates nonstoichiometrically with other interaction partners (30, 39). Nonetheless, our data indicate that the three proteins form a stable 1:1:1 trimer, primarily coordinated *via* high-affinity interactions with NLSs (Fig. 3).

We investigated the nature of intermolecular forces driving the formation of the TPX2–importin-α/β trimer by determining the affinity and stoichiometry with which individual importins interact with TPX2. Importin-αΔIBB (importin-β binding domain removed) was previously shown to bind to TPX2 *via* NLS1 (located at a.a. 284–287) (12, 24). Although NLS1 on TPX2 is well established biochemically, mutating it does not fully abrogate the nuclear import of TPX2 in mammalian cells (35). By measuring the relative apparent K_d_’s of TPX2 truncations and mutants with biolayer interferometry, we discovered the existence of an NLS sequence located at a.a. 123 to 126 of TPX2 (KKLK), termed NLS3. The high dynamic range of biolayer interferometry allowed us to measure both strong interactions (<100 nM) mediated by NLS1 and NLS3 and relatively weak interactions (>400 nM) that persisted in their absence. Finally, NLS3 was previously demonstrated to play a role in nuclear import (35), and our data show that it can mediate TPX2–importin-αΔIBB complex formation in the absence of NLS1 (Fig. 4). Collectively these data suggest that NLS1 and NLS3 on TPX2 may function redundantly to ensure regulation of TPX2.

Canonically, nuclear proteins associate with importin-α/β exclusively through an NLS–importin-α interaction (11). Some proteins, however, functionally interact with importin-β either alone or when it is in complex with importin-α (8). Furthermore, some spindle assembly factors are thought to exclusively interact with importin-β (6, 9) and importin-β alone can inhibit Ran-GTP-mediated microtubule nucleation (36). Nonetheless, biochemical details of spindle assembly factor–importin-β interactions remain uninvestigated. Our data demonstrate that importin-β associates with TPX2 *via* weak interactions, which is reminiscent of other disordered proteins with a different karyopherin–Transportin-1/Karyoperhin-2β (16, 17, 31). Moreover, our data reveal that these weak interactions between TPX2 and importin-β are reversible and promote equilibrium assemblies rather than irreversible aggregation (Fig. 5). In sum, our investigations reveal molecular insight into the TPX2–importin-β complex, which is based on dispersed, weak, reversible interactions. Although such interactions have been implicated in driving condensation (40, 41, 42, 43, 44), we show here that dispersed, weak, reversible interactions are also implicated in preventing it.

Inhibition of TPX2 condensation, and also inhibition of branching microtubule nucleation, by importins displayed a trend opposite to affinity measurements (K_d_ values). Specifically, the hierarchy of inhibition competency is importin-α/β > importin-β >> importin-αΔIBB, whereas the hierarchy of affinity is importin-αΔIBB ≈ importin-α/β >> importin-β (Figs. 1 and 2). This suggests that weak interactions can be a driving force to inhibit condensation and potentially function.

**Figure 6:**
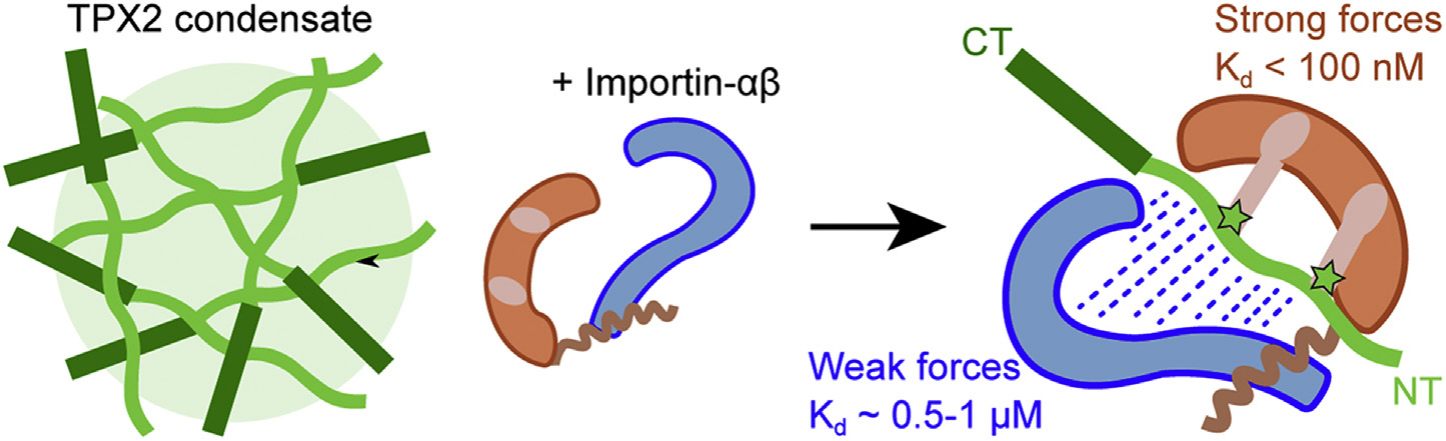
**Schematic of TPX2–importin-α/β tricomplex.** Importin-α association with TPX2 is mediated by a high-affinity interaction with the nuclear localization signals on TPX2, which brings importin-β in the proximity of TPX2. Importin-β interacts with TPX2-NT *via* weak and dispersed forces.

Collectively our data indicate that importin-α acts as a high-affinity bridge that holds TPX2 (*via* its NLS motifs) in proximity to importin-α and importin-β. In so doing both importins appear to collectively suppress TPX2 intermolecular forces and abolish condensation (Fig. 6). We suspect that the primary role of NLS-importin-α high-affinity interactions is to target TPX2 among the many NLS-containing proteins found in a living cell. Once bound to the importin-α/β complex, importin-β (twice as large as importin-α) appears to play a major role in preventing TPX2 condensation through dispersed and relatively weak intermolecular interactions. By elucidating the dual roles of strong and weak interactions in inhibiting TPX2, our study sheds light into how protein phase-separation and spindle assembly are regulated.

## Experimental procedures

Please see the Supplemental information for details. Recombinant proteins are all *Xenopus laevis* versions and were purified to >95% purity (Fig. S4*A*). Size exclusion chromatography (SEC) was carried out on an AKTA Pure-25L. SEC in line with multi-angle light scattering (SEC-MALS) was done with a 3.2/300 column packed with Superdex-200-increase-in line with a Wyatt light scattering machine. Biolayer interferometry was carried out by varying importin concentration and measuring binding to TPX2 that was attached to sensors (Fig. S4*B*), according to vendor specifications of ForteBio. Condensation (phase separation) was achieved by diluting protein mixtures at 0.5 M into 0.1 M salt (KCl). Static and dynamic light scattering measurements were taken at a fixed right angle. Normalized intensity–intensity correlation functions of 20 s duration were collected. To visualize TPX2-mediated microtubule nucleation at indicated excess molar ratios of importins, naturally meiotically arrested *X. laevis* egg cytosol was immunodepleted of endogenous TPX2, indicated proteins were added, and the reaction was imaged over time. Images in each panel are representative crops from a single experimental set with brightness and contrast optimized to allow visualization of the relevant structures. The studies involving *X. laevis* egg cytosol have been approved by the American Association for Laboratory Animal Science (IACUC).

## Data availability

All data are either provided in this article or will be provided upon request to Dr Matthew King, Washington University in St Louis (Matthewking@wustl.edu).

## Supporting information

This article contains supporting information (30, 45, 46, 47, 48, 49, 50, 51).

## Conflict of interest

The authors declare that they have no conflicts of interest with the contents of this article.
