# Supplementary material for: Interaction of spindle assembly factor TPX2 with importins-α/β inhibits protein phase separation
Source: J Biol Chem. 2021 Jul 21;297(3):100998. doi: 10.1016/j.jbc.2021.100998 (PMC8390506; doi:10.1016/j.jbc.2021.100998)
Supplement: Supplemental Figures S1–S4 [file mmc1.docx]

Supplementary Information for

**Interaction of spindle assembly factor TPX2 with importins-α/β inhibit protein phase separation**

**Mohammad S. Safari^a,1^, Matthew R. King^a,d,1^, Clifford P. Brangwynne^b,c^, and Sabine Petry^a,^*****.**

* Correspondence to: Sabine Petry, **Email:**  [spetry@princeton.edu](mailto:spetry@princeton.edu)

**This PDF file includes:**

Supplementary text

Figures S1 to S4

SI References

Supplementary Information Text

**Materials and Methods:**

| **Proteins used** | | |
| --- | --- | --- |
| **Name** | **Details** | **Source** |
| TPX2 | StrHisGFP-TEV-FL_TPX2 (Full Length – aa1-716) | King MR and Petry S., 2020 (30) |
| 3M-TPX2/∆NLS-TPX2 | StrHisGFP-TEV-3M-TPX2 (aa1-716 with K123A, K125A, K126A, K284A, K285A, K327A, K330A) | This paper |
| 2M-TPX2 | StrHisGFP-TEV-2M-TPX2 (aa1-716 with K284A, K285A, K327A, K330A) | This paper |
| TPX2-NT | StrHisGFP-TEV-NT_1-480_TPX2 (N-Terminal aa1-480) | King MR and Petry S., 2020 (30) |
| TPX2-CT | StrHisGFP-TEV-CT_480-716_TPX2 (C-Terminal aa480-716) | King MR and Petry S., 2020 (30) |
| TPX2 aa 1-260 | StrHisGFP-TEV-NT_1-260_TPX2 (N-Terminal aa1-260) | This paper |
| TPX2 aa 1-178 | StrHisGFP-TEV-NT_1-178_TPX2 (N-Terminal aa1-178) | This paper |
| TPX2 aa 1-99 | StrHisGFP-TEV-NT_1-99_TPX2 (N-Terminal aa1-99) | This paper |
| TPX2 aa 1-178∆123 | StrHisGFP-TEV-NT_1-178_TPX2 (N-Terminal aa1-178, with K123A, K125A, K126A) | This paper |
| TPX2 aa 319-716 | StrHisGFP-TEV-CT_480-716_TPX2 (C-Terminal aa319-716) | King MR and Petry S., 2020 (30) |
| importin-β | importin-β (from cleaved from His-GFP-PreScission-importin-β) | This paper |
| importin-α | GST-PreScission-importin-α | This paper |
| importin-α∆IBB | GST-PreScission-importin-α (aa71-end -importin-β Binding domain (IBB) removed) | This paper |
| His-mCherry-EB1 | His-mCherry-End Binding Protein 1 | Thalwani et al., 2018 (46) |
| Cy5-Tubulin | NHS-linked Tubulin (Source: PurSolutions LLC: 032005) | Thalwani et al., 2018 (46) |
| BSA | Bovine Serum Alblumin | Fisher: 23209 |

*E. Coli* strains and growth conditions**:** DH5α cells (New England Biolabs (NEB):C2987I) were used for all subcloning steps. Rosseta2 cells (Fisher: 71-403-4) were used to express proteins for purification. Cells were grown at various volumes in LB Broth (Sigma: L3522), and cells containing importin-α/β constructs were grown in TB media (Sigma: T0918) prepared according to supplier’s instructions. For protein expression, constructs were transformed into Rosseta2 *E. coli* cells and were grown in temperature-controlled incubators shaking at 180 RPM. Cells were grown at 37˚C (0.5-0.7 OD_600_), and induced with 0.75 mM isopropyl-β–D-1- thiogalactopyranoside (IPTG) for another 6 hours at 25˚C. Cell pellets were collected and flash-frozen for future protein purification.

Protein constructs: BSA (Fisher: 23209) and Tubulin (PurSolutions LLC: 032005) are bovine versions and proteins were acquired directly from vendors. Tubulin was labeled with commercial NHS-conjugated dye (Cy5) according to the supplier’s instructions (Sigma: GEPA150101) and its active labeled version purified using a published protocol (45). All remaining proteins are *Xenopus laevis* versions. DNA sequences were sourced from in-house plasmids, the *Xenopus laevis* Gene Collection (Source Biosciences), or synthesized (GenScript).

All TXP2 constructs were cloned as N-terminally tagged Strep6xHisGFP-TEV-TPX2 fusions using a modified pST50 vector (46) and cloned via Gibson assembly (NEB: E2611L). The same vector and cloning strategy were used to generate EB1-mCherry6xHis (47)(26) and His-GFP-PreScission-importin-β. GST-PreScission-importin-α and GST-PreScission-importin-α∆IBB were cloned into a pGEX6P1 vector using Gibson cloning. Insert fragments were PCR amplified from plasmids containing the indicated gene, and in most cases, unmodified from its wild-type sequence. Exceptions are TPX2-2M (StrHisGFP-TEV-2M_TPX2 with K284A, K285A, K327A, K330A), which was custom synthesized (Genscript), and ∆NLS-TPX2 (StrHisGFP-TEV-3M_TPX2 (aa1-716 with K123A, K125A, K126A, K284A, K285A, K327A, K330A) and TPX2 aa1-178∆123 (StrHisGFP-TEV-NT_1-178_TPX2 (N-Terminal aa1-178, with K123A, K125A, K126A), which were both generated using site-directed mutagenesis (Q5® Site-Directed Mutagenesis Kit, NEB, E0554S). All constructs were fully sequenced and confirmed to have no errors.

Protein purification: For all TPX2 constructs, a previously published purification scheme was used (30). Briefly, cells were lysed using an EmulsiFlex (Avestin) in lysis buffer (0.05M Tris-HCl, 0.015M Imidazole, 0.75M NaCl, pH 7.75) containing 0.0002M phenylmethylsulfonyl fluoride (PMSF), 0.006M β-mercaptoethanol (βME), cOmplete™ EDTA-free Protease Inhibitor tablet (Sigma 5056489001), and 1000U Dnase I (Sigma 04716728001). The lysate was clarified, bound to Ni-NTA agarose beads (Qiagen 1018236), washed, and eluted in lysis buffer containing 200mM Imidazole. Protein was further purified via gel filtration (Superdex 200 HiLoad 16/600, GE Healthcare – 28-9893-35) in CSF-XB buffer (0.01M Hepes, 0.002M MgCl_2_, 0.0001M CaCl_2_, 0.004M Ethylene glycol-bis(2-aminoethylether)-N,N,N′,N′-tetra-acetic acid (EGTA), 10% w/v sucrose, pH-7.75) containing either 0.1M KCl for extract assays or 0.5M KCl for condensate assays.

GST-importin-α clarified lysates were prepared in the same way with the exception of the lysis buffer (0.05M Na_2_HPO_4_/NaH_2_PO_4_, 0.5M NaCl, 5 mM β-mercaptoethanol, pH=7.45) and they were bound to a GST affinity column ([GSTrap™ Fast Flow](https://www.sigmaaldrich.com/catalog/product/sigma/ge17513102?lang=en&region=US), GE Healthcare: 17-5131-02). The column was washed (0.05M Na_2_HPO_4_/NaH_2_PO_4_, 0.5M NaCl, 5 mM β-mercaptoethanol, pH=7.45), protein was eluted (0.05M Na_2_HPO_4_/NaH_2_PO_4_, 0.5M NaCl, 5 mM β-mercaptoethanol, 100 mM Glutathione-reduced, pH=7.45) and peak fractions were pooled.

His-GFP- PreScission -importin-β cells were lysed as explained above (Binding buffer: 50 mM Tris, 750 mM NaCl, 10 mM β-mercaptoethanol, 2% glycerol, pH = 7.88) and spun at 27000 rpm for 30 minutes. The protein was further purified from the lysed supernatant via a NiNTA affinity purification using a His-Prep FF 16/10 column (Column Volume ~ 20 ml). The column was then washed with 10 column volumes (CV) of binding buffer and the bound importin-β was eluted with a sharp Imidazole gradient (4 CV). The eluted fractions (~25 ml) were concentrated using an Amicon 50 kDa membrane to 10 ml. 500 μl of 6xHis-HRV-3C protease at 2 mg/ml were added to 10 ml protein solution (1:50 w/w; importin-β concentration was measured by absorbance at 280 nm using Nanodrop and the protein purity after His-purification was estimated to be 90%) and the sample dialyzed with a 20 kD cut-off against 4L of 25 mM HEPES, 150 mM NaCl, 5 mM β-mercaptoethanol, 5% glycerol, pH = 7.75.

After at least 16 h, the protein was taken out ,concentrated to 4-5ml, use for size-exclusion chromatography (SEC) on a HiLoad Superdex 200-increase column (16/600) equilibrated in buffer (50 mM Tris, 100 mM NaCl, 10 mM β-mercaptoethanol, pH = 7.5). Fractions containing cleaved importin-β were pooled and concentrated to 500 ul, which was subjected to a final affinity purification to remove the protease in a His-Trap high performance (5 ml) column. The flow-through with purity >96% was collected and concentrated to 100 mg/ml in CSF-XB containing 0.1M KCl and 10% Sucrose (pH = 7.85).

EB1-6xHis-mCherry was purified as described previously (26). All proteins were aliquoted into single-use volumes, flash-frozen, and stored at -80˚C. Before use, all proteins were thawed on ice and pre-cleared of aggregates via ultracentrifugation at 80,000 RPM for 15min in a TLA100 rotor in an Optima MAX-XP ultracentrifuge at 4˚C. Protein concentrations were determined by OD280 absorbance measured via nanodrop (model ND-1000) using the extinction coefficient of the proteins (48).

Size-Exclusion chromatography in line with Multi-Angle Light Scattering (SEC-MALS): To achieve the highest resolution and separation of protein complexes, Superdex-200-increase-3.2/300 was equilibrated in low salt CSF buffer with no crowder (0.01M HEPES, 0.002M MgCl_2_, 0.0001M CaCl_2_, 0.004M Ethylene glycol-bis(2-aminoethylether)-N, N, N′, N′-tetraacetic acid (EGTA), 6 mM β-mercaptoethanol, 0.1M KCl, pH-7.75) in line with a Wyatt scattering instrument operating with a 632.38 nm red laser and 18 detectors located every 20° to monitor scattered light at multiple angles. The flow rate was 0.04 ml/min and a 25 μl sample loop was used for injection. The molecular weights of the eluted complex were further characterized by a Debye plot using Kc/R(θ) = 1/M_w_ + B_2_c (44). Prior to injection into the column, all the buffers and protein solutions were filtered with 0.22μm PTFE filters (hold up volume <10 μl, product number SLGV004SL from Millipore Sigma). 60 μM stock TPX2 in high salt CSF-XB containing 0.5 M KCl and 10% Sucrose was diluted to a final of 2 μM in low salt CSF buffer, containing 0.1 M KCl and no sucrose, and the sample (final salt of 0.17M KCl) was injected to the column. To monitor the association of TPX2 to importins, the GFP-TPX2 concentration was fixed at 2μM and importins were added to a final concentration of 20μM (final salt of 0.17M KCl) prior to sample injection. Control samples containing only importins were injected at 20 μM in CSF containing 0.1M KCl and no sucrose.

Analytical Size Exclusion to test the reversibility of TPX2 importin-β Complex: To test the reversibility of TPX2-importin-β complex, we conducted size exclusion chromatography with a constant TPX2 concentration of 2 μM while varying importin-β concentration ranging from 2 to 30 μM. A Superdex200-increase-3.2/300 column was equilibrated in CSF buffer containing 0.1 KCl, 6 mM β-mercaptoethanol and no sucrose, and the sample was injected as described above.

Biolayer interferometry (OCTET): Binding kinetic measurements were performed using biolayer interferometry via an Octet instrument. Anti-Penta-His (HIS1K) sensors were purchased from ForteBio. The sensors were washed in CSF containing 0.5M KCl and no sucrose. The TPX2 or TPX2 fragments containing 6xHis-tags were loaded into the sensor for 30-45 seconds at a low concentration of 120 nM to ensure no aggregation occurs on the sensor surface. The sensor was then washed in low salt CSF containing 0.1 M KCl and no sucrose for 300 seconds prior to exposure to importin solutions. Equilibrium binding was performed in wells with varying concentrations of importins, for 900 seconds to ensure the binding curve was plateaued. At longer time scales, the association and dissociation rate of ligand reaches the equilibrium. Using a simple titration curve, the amplitude can be plotted as a function of ligand concentration. Upon equilibrium, the response follows the equation $R=R_{max}C/(K_{d}+C),$where R_max_ is the maximum plateau, and K_d_ is the equilibrium dissociation constant (49). The height of the binding amplitude was obtained by subtracting the signal from the control sample with no importin. It was essential to include a control sample for every run, as the loading of TPX2 constructs on the sensor can vary. Each data was replicated in two distinct measurements and the errors were calculated. For importin-β binding assays, 0.01% (v/v) tween-20 was used in the buffer to eliminate non-specific binding to sensors. All the importin-α-ΔIBB data were collected in regular CSF buffer containing 0.1M KCl, no tween, and no sucrose.

Condensation (phase separation) assay: Proteins were diluted to reach a 5x final concentration in a CSF-XB Buffer containing 500 mM KCl salt at 4˚C, then diluted 1:4 in CSF-XB containing no salt at room temperature (23˚C) to reach a final salt concentration of 100mM. To image condensates via epifluorescence microscopy, the reaction mixture was immediately pipetted into a flow chamber (constructed with double-stick tape, a glass slide, and a 22x22mm coverslip). The slide was placed coverslip-side down into a humidity chamber for 10 minutes at room temperature to allow condensates to settle; the reaction was then imaged via epi-fluorescent microscopy. Crowding agents were never used.

Static and Dynamic Light Scattering (SLS and DLS): Light scattering measurements were performed with a Wyatt instrument operating with red laser (λ = 632.8 nm), with the detector fixed at 90° to monitor the scattered intensity. Protein samples were filtered with 0.22 μm filters (hold up volume <10ul) prior to measurements. Normalized intensity-intensity correlation functions $g_{2}$(*q,*τ) of 20 second duration were recorded. We fitted the intensity-intensity correlation functions possessing one broad decaying exponent using the cumulant function(50):

$$\boldsymbol{g}_{\boldsymbol{2}}\left( \boldsymbol{q,\tau} \right)\boldsymbol{-1=}\left( \boldsymbol{A}\exp\left( \boldsymbol{-}\frac{}{\boldsymbol{\tau}_{\boldsymbol{m}}} \right)\boldsymbol{(1+\mu}\boldsymbol{\tau}^{\boldsymbol{2}}\boldsymbol{/2)} \right)^{\boldsymbol{2}}\boldsymbol{+ ԑ}\left( \right)$$

where *A* is the scattering amplitude, $\boldsymbol{ԑ}\left( \boldsymbol{\tau} \right)$ accounts for any noise in the signal, and $\boldsymbol{\mu}\boldsymbol{\tau}^{\boldsymbol{2}}$ is the polydispersity index. In our measurements, $\boldsymbol{ԑ}$ was one or two orders of magnitude lower than the amplitudes. For$\boldsymbol{g}_{\boldsymbol{2}}$s possessing two exponent decays, we fitted the data with two relaxation times corresponding to monomer/oligomers and condensates, respectively.

$$\boldsymbol{g}_{\boldsymbol{2}}\left( \boldsymbol{q,\tau} \right)\boldsymbol{-1=}\left( \boldsymbol{A}_{\boldsymbol{m}}\exp\left( \boldsymbol{-}\frac{}{\boldsymbol{\tau}_{\boldsymbol{m}}} \right)\boldsymbol{+}\boldsymbol{A}_{\boldsymbol{c}}\mathbf{exp}\left( \boldsymbol{-}\frac{}{\boldsymbol{\tau}_{\boldsymbol{c}}} \right) \right)^{\boldsymbol{2}}\boldsymbol{+ ԑ}\left( \right)$$

We used $\boldsymbol{\tau}_{\boldsymbol{m}}$ to determine the average diffusivity, *D_m_*, from *D_m_* = (*q^2^*$\boldsymbol{\tau}$)^–1^, where *q* = $\frac{\mathbf{4}\boldsymbol{\pi n}}{\boldsymbol{\lambda}}\mathbf{sin(}\boldsymbol{\theta}\mathbf{/2)}$ is the wave vector at a scattering angle of 90^o^, λ = 632.8 nm is the wavelength of the incident red laser and *n* = 1.33 is the solution refractive index.

In addition, we tested the same samples with DLS to monitor the importin ratio at which no sub-diffraction condensate was detected.

***Xenopus laevis* egg cytosol assays**

Cytosol preparation**:** *Xenopus laevis* cytosol naturally arrested in meiosis II was prepared as described in (51). Briefly, *Xenopus laevis* eggs were collected after an overnight laying period. Eggs from individual frogs were kept separate but prepared in parallel, and typically 2 batches of eggs were used. Egg jelly coats were removed, and cytosol was fractionated away from egg yolk, membranes, nucleus, and organelles by centrifugation (10200 RPM in HB-6 for 15 minutes). Eggs were constantly maintained at 18˚C via preparation in a temperature-controlled room. Undiluted cytosol was collected, supplemented with Cytochalasin-D, protease inhibitors, ATP, and creatine phosphate, and kept at 4˚C until use.

*Xenopus* cytosol was immunodepleted of endogenous TPX2 as described in (30). Briefly, immunoaffinity purified antibodies against TPX2 or an unspecific IgG control antibody were conjugated to magnetic Dynabeads Protein A (ThermoFisher: 1002D) at 4˚C overnight. Antibody-conjugated beads were split into two equal volume aliquots; the supernatant was removed from one aliquot using a magnetic block and *Xenopus* cytosol was added. Beads were gently suspended in cytosol every 10 minutes for 40 minutes. Cytosol was removed from beads (using magnetic block) and then subjected to another round of depletion using the same procedure with the second aliquot of antibody-conjugated beads. Immunodepletion was assessed via Western blots and functional assays.

Branching microtubule nucleation assay: microtubule nucleation reactions were carried out as described in (30). Briefly, *Xenopus* cytosol was supplemented with fluorescently labeled tubulin ([1 µM] final) to visualize microtubules, mCherry-fused End Binding protein 1 (EB1) ([100 nM] final) to track microtubule plus ends, and sodium orthovanadate ([0.5 µM] final) to inhibit dynein-mediated microtubule gliding. Mono-dispersed purified TPX2 (with or with our importins) was added at specified concentrations and excess molar ratios. In all experiments, CSF-XB buffer containing 0.1 M KCl was used to match total dilution across all experiments (25% of extract volume). All reagents used were in CSF-XB buffer containing 0.1 M KCl. The reaction mixture was prepared on ice, then pipetted into a coverslip flow chamber at 18˚C, which marked the start of the reaction.

Each reaction was imaged via TIRF microscopy for 30-40 minutes at 10-20 second intervals. Typically, two reactions (e.g. 0x and 1x excess importin-β) were prepared and flown into parallel chambers onto a single slide, and each was imaged every 10-20 seconds by alternating between fields using the ‘XY acquisition’ function in NIS Elements (Nikon). This procedure enabled assessing multiple reactions within single cytosol preparation that has a finite lifetime (~2-6 hours). All experiments using *Xenopus* egg cytosol were reproduced at least three times using separate cytosol preparations. Similar results were seen in all replicates.

Analysis of branching microtubule nucleation: Total microtubule number per reaction was determined by counting the number of EB1 spots on microtubule plus ends in the entire field of view. EB1 detection was achieved via the ‘Color Threshold’ function (Otsu threshold) and the ‘Particle Analyzer’ function (size 0.1 -1 µm^2^) on FIJI. Parameters were optimized for each data-set according to visual assessment of tracking accuracy. Plots were generated by plotting the normalized number of EB1 detections (relative to 0x importins condition) for the 20-minute frame for each reaction.

Image collection and processing: The imaging technique used is indicated in each corresponding figure legend. Total Internal Reflection Fluorescence (TIRF) and Epifluorescence (Epi), microscopy methods were carried out on a Nikon TiE microscope with a 100X, 1.49NA oil immersion objective and an Andor Zyla sCMOS camera. All experiments were carried out at 18˚C in a temperature-controlled room.

NIS-Elements software was used for all image acquisition. All images within a data set were taken with identical imaging parameters. Binning (2x2) was used in the case of *Xenopus* cytosol branching microtubule nucleation assays, but not for phase separation assays.

FIJI was used for all image analyses. Images of branching microtubule nucleation are 500 by 500-pixel crops of the center of the imaging field (comprising ¼ the area of the total field). Images of GFP-TPX2 phase separation are 100 by 100-pixel crops of a representative field. All images were processed using optimal brightness and contrast windows for each field to allow visualization of the relevant structure. Max/min windows between samples did not deviate more than 2-fold. Images within a figure panel were acquired and processed in the same setting.

Adobe Illustrator/Inkscape was used to generate illustrations and compile figures. Oracle was used to generate all plots. The surface electrostatic potential and surface hydrophobic residues were visualized by Pymol for importin-β (PDB: 1QGK) and importin-αΔIBB (PDB: 4U5L).


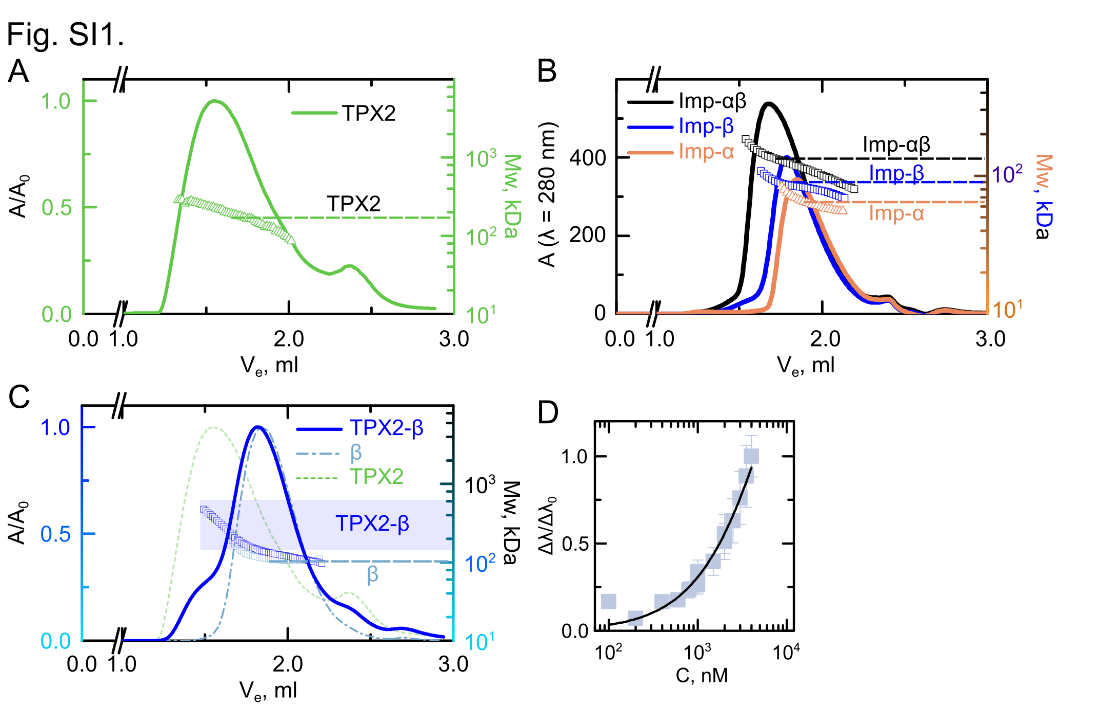


Fig. S1. Characterization of importin-α/β heterodimer and TPX2-importin-β complex. (*A*) SEC-MALS elution profile of GFP-TPX2. The MALS profile indicates that GFP-TPX2 is monodisperse and mainly contains monomers. (*B*) SEC-MALS elution profile of GST-FL-importin- α (light brown), importin-β (blue), and GST-importin-α:importin-β (black). The arrows indicate the molecular weights of 63.6 kDa, 83.7 kDa, 130.0 kDa for GST-importin- α, importin-β, and GST-importin-α:importin-β respectively. (*C*) SEC-MALS elution profile of TPX2-importin-β complex (dark blue) and importin-β alone (light dashed blue). TPX2:importin-β MALS profile suggests there is a population of oligomers eluting from 1.2-1.6ml with molecular weights ranging from 200-700 kDa. (*D*) Normalized BLI-Octet binding curve for CT-TPX2 (319-716) and importin-β.


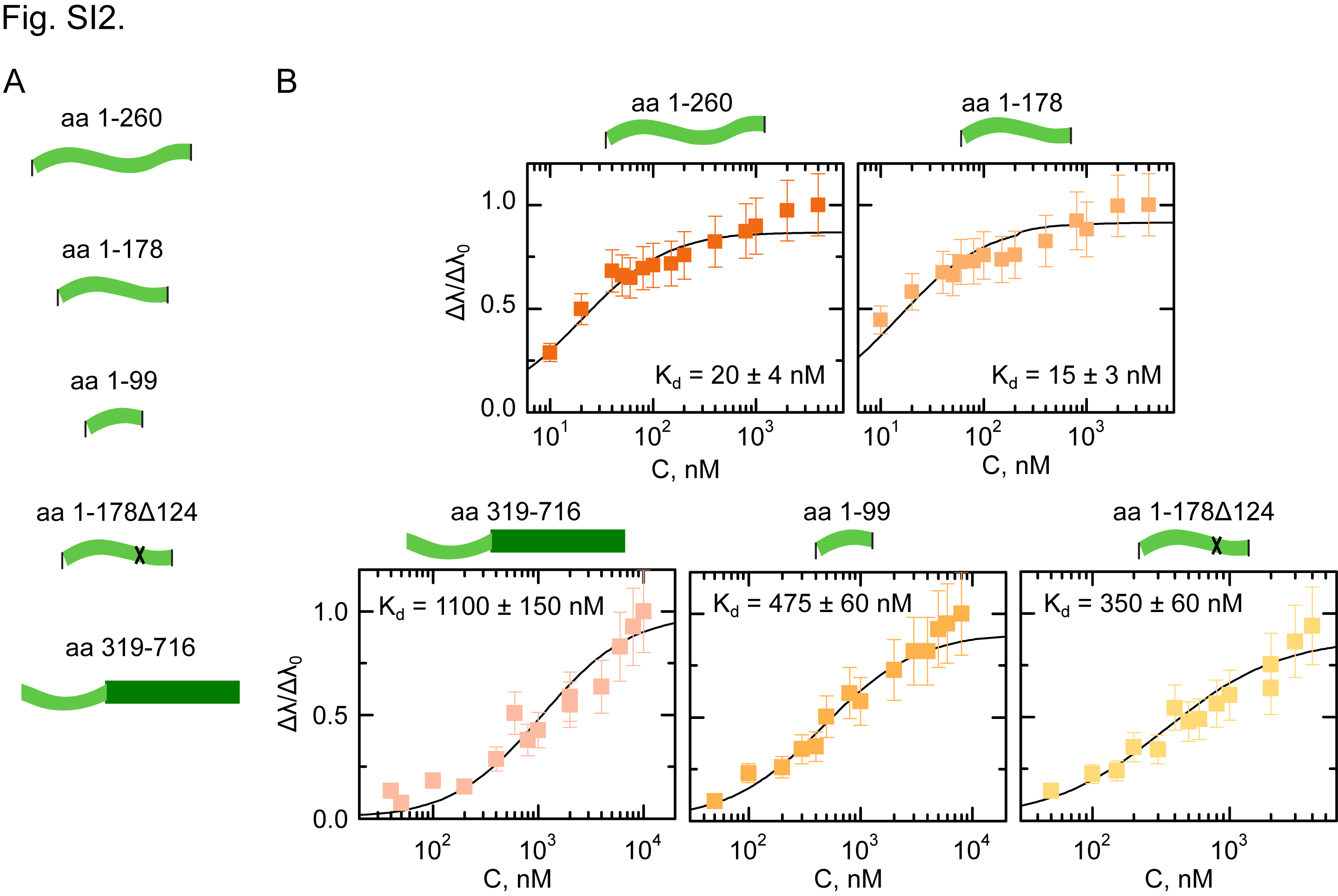


Fig. S2. Identification of TPX2 putative NLS site aa123-126. (*A*) Schematics of tested TPX2 constructs for binding to importin-α∆IBB (*B*) Bio-layer interferometry binding curves for indicated TPX2 constructs with importin-α∆IBB.


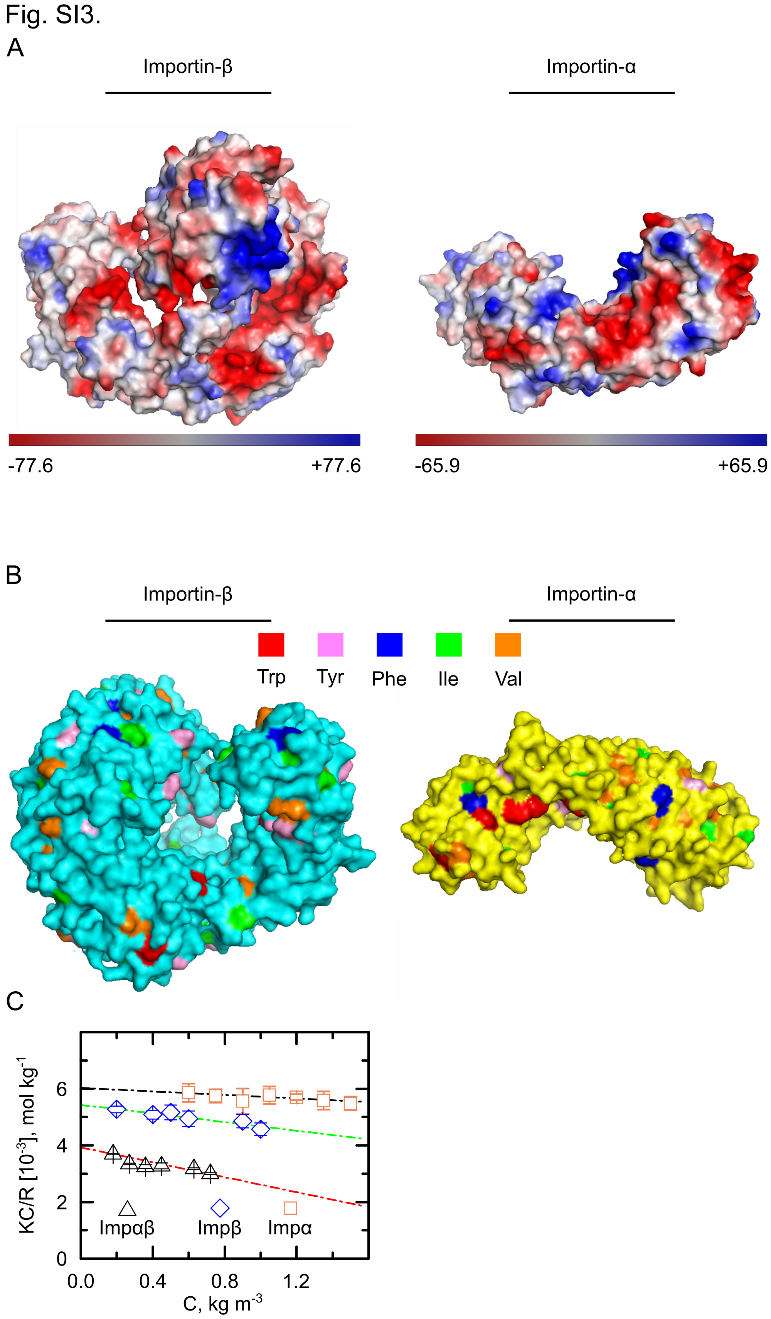


Fig. S3. Illustration of exposed electrostatic potential and hydrophobic patches on importin surfaces. (*A*) Surface electrostatic potential of importin-β and importin-αΔIBB. The net charge of Importin-β and importin-αΔIBB are -38 and -9 respectively. The red and blue indicate negative and positive patches, respectively. (*B*) Exposed hydrophobic patches on the surface of importins. The residues are colored as Tryptophan (red), Tyrosine (pink), Phenylalanine (blue), Iso-leucine (green), and Valine (orange). (*C*) The osmotic compressibility of the importin.


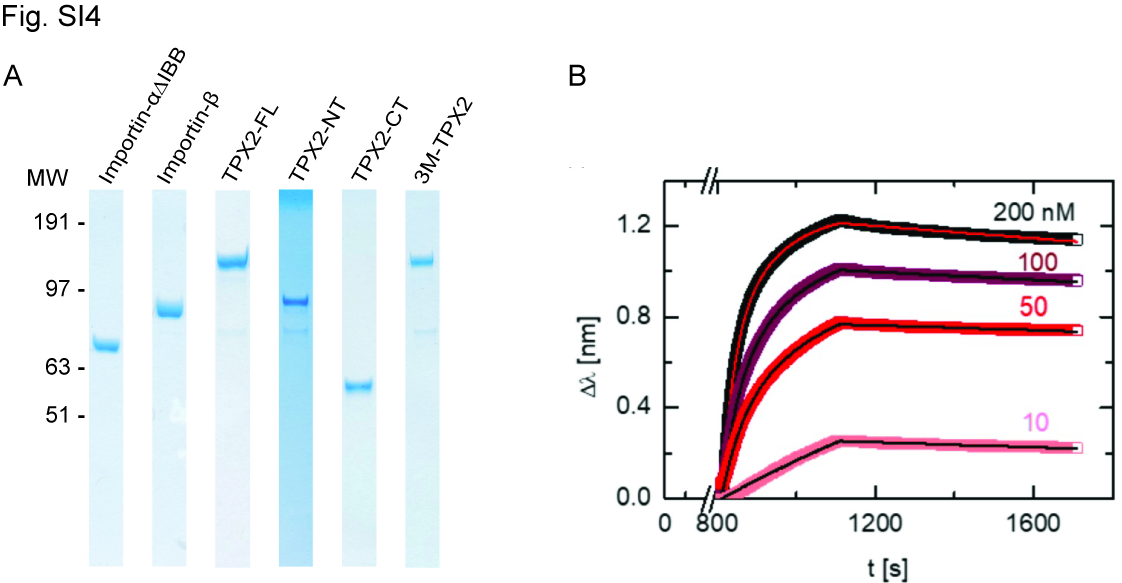


Fig. S4. Representative biolayer interferometry data and protein gels (*A*) Raw OCTET data for association and dissociation of the TPX2-loaded sensor to importin-αβ heterodimer at four concentrations of 10, 50, 100, and 200 nM, respectively. (*B*) Representative gels of the proteins used in this study. Shown are importin-α∆IBB (GST-PreScission-importin-α∆IBB); Importin-β (importin-β cleaved from His-GFP-PreScission-importin-β);TPX2-FL (StrHisGFP-TEV-TPX2-Full Length - aa1-716); TPX2-NT (StrHisGFP-TEV-NT_1-480_TPX2 - N-Terminal aa1-480); TPX2-CT (StrHisGFP-TEV-CT_480-716_TPX2 -C-Terminal aa480-716); 3M-TPX2 (StrHisGFP-TEV-3M_TPX2 (aa1-716 with K123A, K125A, K126A, K284A, K285A, K327A, K330A)

**SI References**

30. M. R. King, S. Petry, Phase separation of TPX2 enhances and spatially coordinates microtubule nucleation. *Nat. Commun.* **11**, 1–13 (2020).

45. A. Hyman, *et al.*, Preparation of modified tubulins. *Methods Enzymol.* **196**, 478–485 (1991).

46. S. Tan, R. C. Kern, W. Selleck, The pST44 polycistronic expression system for producing protein complexes in Escherichia coli. *Protein Expr. Purif.* **40**, 385–395 (2005).

47. A. Thawani, R. S. Kadzik, S. Petry, XMAP215 is a microtubule nucleation factor that functions synergistically with the γ-tubulin ring complex. *Nat. Cell Biol.* **20**, 575–585 (2018).

48. C. Gill, P. H. Von Hippel, <Gill$vonHippel1998-proteinexcoeff-319.pdf>. **326**, 319–326 (1989).

49. , SPR nonlinear kinetics.pdf.

50. B. J. Frisken, dynamic light-scattering data. **40**, 4087–4091 (2001).

51. E. Hannak, R. Heald, Investigating mitotic spindle assembly and function in vitro using Xenopus laevis egg extracts. *Nat. Protoc.* **1**, 2305–2314 (2006).
